# Methods to Calculate Entropy Generation

**DOI:** 10.3390/e26030237

**Published:** 2024-03-07

**Authors:** Jude A. Osara, Michael D. Bryant

**Affiliations:** 1 Surface Technology and Tribology, Department of Mechanics of Solids, Surfaces and Systems, University of Twente, 7522 NB Enschede, The Netherlands; 2 Mechanical Engineering Department, University of Texas at Austin, Austin, TX 78712, USA; michael.bryant@machineessence.com; 3 Machine Essence Corporation, Austin, TX 78746, USA

**Keywords:** entropy generation, non-equilibrium thermodynamics, phenomenology, second law, thermodynamic potentials

## Abstract

Entropy generation, formulated by combining the first and second laws of thermodynamics with an appropriate thermodynamic potential, emerges as the difference between a phenomenological entropy function and a reversible entropy function. The phenomenological entropy function is evaluated over an irreversible path through thermodynamic state space via real-time measurements of thermodynamic states. The reversible entropy function is calculated along an ideal reversible path through the same state space. Entropy generation models for various classes of systems—thermal, externally loaded, internally reactive, open and closed—are developed via selection of suitable thermodynamic potentials. Here we simplify thermodynamic principles to specify convenient and consistently accurate system governing equations and characterization models. The formulations introduce a new and universal Phenomenological Entropy Generation (PEG) theorem. The systems and methods presented—and demonstrated on frictional wear, grease degradation, battery charging and discharging, metal fatigue and pump flow—can be used for design, analysis, and support of diagnostic monitoring and optimization.

## 1. Introduction

System analyses based on energy conservation alone have been shown inadequate for consistent characterization of real, often nonlinear, system transformation. The introduction of the second law via the works of Carnot, Clapeyron [1], Clausius, Massieu [2] and others established thermodynamics as a field for consistent description of changes in a system undergoing any form of energy conversion. For centuries, classical thermodynamics has been restricted to equilibrium and near-equilibrium transformations. Defining a minimum condition for a system to exist or process to occur in nature, Rayleigh’s dissipation function [3], Onsager’s least energy dissipation [4] (an application of his reciprocal relations of microscopic reversibility) and Prigogine’s minimum entropy generation [5,6]—similar statements expressed as $\delta S^{\prime }=YdX/T$—set the stage for thermodynamic characterization of real system transformation, a field commonly known as irreversible thermodynamics. Here, $\delta S^{\prime }$ is entropy generation, *Y* is generalized force, *dX* is generalized displacement and *T* is temperature. The correlation between energy dissipation (or entropy generation) and system degradation—advanced and permanent disorganization of material structure—has been theorized and experimentally verified. Recently, several multi-disciplinary system characterizations have emerged, presenting experimental results consistent with entropy-based formulations. These works show high accuracies in analyzing active system transformations, with inconsistencies attributable to the often-used “steady state” assumption. While some long-running systems operate predominantly in pseudo-steady state, e.g., very high-cycle fatigue of steels, most loaded systems often transform unsteadily, limiting the applicability of energy conservation and steady-state entropy characterizations. Recent works by Osara and Bryant [7,8,9,10] using the Degradation-Entropy Generation (DEG) methodology [11] to assess battery degradation, grease degradation and metal fatigue, showed a near 100% accurate and consistent characterization of these systems undergoing severely abusive loads. DEG methods relate increments of degradation to increments of entropy generation. Instead of a “steady state” assumption, Osara and Bryant [7,8,9,10] augmented laws of thermodynamics with the thermodynamic potentials to formulate entropy generation. Methods to calculate entropy generation under general conditions for all systems are needed to enable real-time assessment of system transformation.

This article will develop entropy generation $\delta S^{\prime }$ for **open and closed systems** as the difference

(1a) $$\delta S^{\prime }={\delta S}_{phen}-{dS}_{rev}\geq 0$$

between a phenomenological entropy generation function ${\delta S}_{phen}$, evaluated via suitable measurements of variables over an irreversible path in a thermodynamic state space between initial and final (or current) thermodynamic states; and a reversible entropy function ${dS}_{rev}$, evaluated over a reversible path in the same state space between the same initial and final states. The thermodynamic state space consists of any independent variables that characterize the thermodynamic state and all active irreversible dissipative processes. The reversible path for ${dS}_{rev}$, which is the projection of the irreversible *phenomenological* path of ${\delta S}_{phen}$ onto the reversible subspace, consists of the thermodynamic state variables of the phenomenological path sans the irreversible dissipative process variables. This approach will eliminate many tenuous “steady state” assumptions and loopholes in existing approaches. To obtain entropy generation $S^{\prime }$, Equation (1a) must be integrated between the initial and final/current states, with entropy functions ${\delta S}_{phen}$ and ${dS}_{rev}$ integrated over their respective paths. True reversibility excludes process rates and time effects, hence, the reversible path defined herein marks the theoretical limit of a real-time-based process via a linear function joining the initial and final states.

### 1.1. Local Equilibrium

Prigogine posited: given that true equilibrium is asymptotic for all real systems, every continuous macroscopic system is made up of elements for which observable state properties (such as temperature and pressure) can be instantaneously determined or measured, thereby rendering equilibrium formulations describing these properties valid for each element in the macrosystem. Each element is, therefore, in local equilibrium [5,6,12,13]. This theorem allows the extension of reversibility-based formulations to real systems, with entropy generation representation. While the system variables are spatial and temporal functions, many real systems operate with near-uniform internal properties, primarily changing with time.

### 1.2. States, Paths and Path (Line) Integrals

The following principles will be judicious to Section 2:

1. Thermodynamics often involves changes in variables between two states. Variables include a set of the independent thermodynamic state variables ***Z*** chosen and measured for a particular system, and state dependent system properties which are functions of the states of ***Z***. The ***Z*** characterize a system’s thermodynamic state and can include temperature *T*, pressure *P*, and number of moles *N*, among others. Changes in system properties such as energy *E*, entropy *S*, temperature *T* and $S_{rev}$ use the exact differential *d*; are path independent, wherein changes in properties over an irreversible path (*irr*) are identical to changes in properties over a reversible path (*rev*), e.g., *dE* = *dE_irr_ = dE_rev_*; and the line integral $\int_{o}^{f}dE=∆E=E_{f}-E_{o}$ depends only on the property values at the beginning and end states (*o* and *f*). This is the thermodynamic state principle.
2. Path-dependent variables such as work *W*, heat transfer Q, entropy generation $S^{\prime }$ and $S_{phen}$ depend on what occurs along the path between states *o* and *f*, and use the inexact differential δ such that $\int_{o}^{f}\delta W=W$ must be accumulated over all instants of time *t* along the (assumed known) transformation path between times $t_{o}$ and $t_{f}$. The path-dependent parameters will depend on a set of variables Z = {***Z***$,\zeta_{k}$} assumed to be time dependent, observable, and measurable. The ***Z*** characterize the thermodynamic state, whereas the $\zeta_{k}$ characterize any active irreversible dissipative processes. Via a suitable numerical integration such as the trapezoid rule, with the $Z\left(t\right)$ = {***Z***(*t*), $\zeta_{k}\left(t\right)$} measured as points $Z\left(t_{j}\right)\;$= {***Z***$\left(t_{j}\right)$, $\zeta_{k}(t_{j})\}$ suitably spaced at time instants $t_{o}<$ $t_{j}$ < $t_{f}$ in accord with the sampling theorem [14], the increments δW can be accumulated into the line integral $\int_{o}^{f}\delta W=W$.
3. Exact differentials dE, state functions of the independent state variables ***Z***(*t*), if intractable, can be numerically integrated over the (ideal) reversible path per methods of the prior paragraph. The reversible path must transit states *o* to *f* in quasi-equilibrium and be continuous and maximally smooth over time, which can be approximated by linear functions with slope determined by the end states, for example, if $dE\left(t\right)=dE(Z(t))$, components of ***Z***(*t*) with slope $\frac{{Z(t}_{f})-{Z(t}_{o})}{t_{f}-t_{o}}$ where ${Z(t}_{f})$ and ${Z(t}_{o})$ must be measured or known at the beginning and final times $t_{o}$ and $t_{f}$. With this, the line integral $\int_{o}^{f}dE=\int_{t_{o}}^{t_{f}}\frac{dE}{dt}dt=\int_{t_{o}}^{t_{f}}\sum_{Z}\frac{dE}{dZ}\frac{dZ}{dt}dt=\sum_{Z}\frac{{Z(t}_{f})-{Z(t}_{o})}{t_{f}-t_{o}}\int_{t_{o}}^{t_{f}}\frac{dE}{dZ}dt$, where sum index *Z* denotes a sum over all the components of ***Z***, and *dE*/*dZ* must be evaluated at each time instant $t_{o}<$ $t_{j}$ < $t_{f}$ along the reversible path.
4. For reversible processes ${dS}_{rev}$ with initial and final states ${{dS}_{rev}}_{o}$ and ${{dS}_{rev}}_{f}$, ${dS}_{rev}(t)=\frac{{{dS}_{rev}}_{f}-{{dS}_{rev}}_{o}}{t_{f}-t_{o}}(t-t_{o})+{{dS}_{rev}}_{o}$ satisfies the reversible path approximations of item 3.
5. A phenomenological path (*phen*) through the thermodynamic state space enclosing Z = {***Z***$,\zeta_{k}$} defined in item 2 includes nonzero $\zeta_{k}\left(t\right)$. A reversible path (*rev*) in the reversible subspace {***Z***} of Z [15] involves only the thermodynamic states ***Z_rev_***, not the $\zeta_{k}$. The projection of the set of points $Z\left(t_{j}\right)\;$= {***Z***$\left(t_{j}\right)$, $\zeta_{k}(t_{j})\}$ that comprise an irreversible *phenomenological* path onto the reversible subspace is the set of points {$Z_{rev}\left(t_{j}\right)\}$ [15]. The system inputs, active mechanisms and dynamics are determined by the phenomenological path.

These principles are based on classical approaches [2,16,17]. While classical theories are limited to equilibrium and near-equilibrium states, we here introduce concurrent physically observable states to characterize real, often highly dissipative, far-from-equilibrium systems.

## 2. Irreversible Thermodynamics and Entropy Generation

### 2.1. Combining Internal Energy and Entropy Balances

For a stationary thermodynamic system (open and closed), excluding gravitational effects, the first law of thermodynamics

(2) $$dU=\sum \delta Q-\sum \delta W+\sum {\left(u+Pv\right)dN}_{e}+\sum \mu_{k}dN_{k},$$

balances dU, the change in overall system “internal” energy. Here ∑δQ is the sum of all heat exchanges across the system boundary, ∑δW is the sum of all work transfers across the system boundary, $\sum {\left(u+Pv\right)dN}_{e}$ is the sum of energy transfers by matter flows ${dN}_{e}$ across the system boundary (for open systems only), and $\sum \mu_{k}dN_{k}$ is the sum of all compositional energy changes within the system boundary. Embedded in the compositional change term $\sum \mu_{k}dN_{k}$ are, in terms of mole number $N_{k}$ of species *k*, changes in the quantity of matter due to chemical reactions ${{dN}^{r}}_{k}$ and ionic mass diffusions within the system boundary ${{dN}^{d}}_{k}$, i.e., $dN_{k}={{dN}^{r}}_{k}+{{dN}^{d}}_{k}$, where ${N^{r}}_{k}$ and ${N^{d}}_{k}$ are reactive and internally diffusive species, respectively. Also, *u* is molar internal energy, *P* is pressure, *v* is molar volume and μ is chemical potential. The product *Pv* is termed the flow work in open systems. Via the molar mass, Equation (2) can be re-written in terms of mass *m*. Inexact differential δ indicates path-dependent variables.

A statement of the second law—the Clausius inequality—gives the change in entropy of a **closed system** as $dS\geq \frac{\delta Q}{T}$ where *δQ/T* is entropy flow by heat transfer, which can be positive or negative depending on the transfer direction, and *T* is the temperature of the boundary where the energy/entropy transfer takes place. Via the thermodynamic state principle—item 1 of Section 1.2—and the entropy balance [5,6,12], entropy change accompanying an **open system** process can be evaluated along a real and often nonlinear **irreversible path (*irr*) as**

(3) $$dS=dS_{irr}=\frac{\delta Q}{T}+\frac{\left(u+Pv\right){dN}_{e}}{T}+\delta S^{\prime }={\delta S}_{Q}+{dS}_{N_{e}}+\delta S^{\prime },$$

where ${\delta S}_{Q}=\frac{\delta Q}{T}$ is entropy transfer via heat, ${dS}_{N_{e}}=\frac{\left(u+Pv\right){dN}_{e}}{T}$ is entropy transfer via mass, and $\delta S^{\prime }$ is internal entropy generation which always accompanies permanent change and structural disorganization of the evolving system. While *dS*, ${\delta S}_{Q}$ and ${dS}_{N_{e}}$ can be positive or negative, the second law asserts $\delta S^{\prime }\geq 0$. Without a priori knowledge of the entropy generation, change along the irreversible path, Equation (3), cannot be determined. Along a **reversible *rev* (ideal and linear) path** with $\delta S^{\prime }=0$,

(4) $$dS=dS_{rev}=\frac{\delta Q_{rev}}{T}+\frac{{\left(u+Pv\right)}_{rev}{dN}_{e}}{T}={\delta S}_{Q,rev}+{dS}_{N_{e},rev}.$$

Substituting $\delta W=\sum Y_{l}dX_{l}\;$ [18] for external/boundary works including compression PdV, strain work Vσdε, electrical work νdq and others into Equation (2) and combining with entropy Equation (3) gives the combined first and second laws as

(5) $$T\delta S^{\prime }+dU=TdS-\sum Y_{l}dX_{l}\;+\sum \mu_{k}dN_{k}=T{\delta S}_{U,phen}(S,X_{l},N_{k}),$$

where *Y_l_* are intensive variables such as pressure *P*, strength/stress σ, voltage ν, etc.; $X_{l}$ are the system’s extensive variables such as volume *V*, strain ε, charge q; and $T{\delta S}_{U}\left(S,X_{l},N_{k}\right)$, a path-dependent inexact differential, is defined by and equal to the middle expression of Equation (5). **The script notation distinguishes** $S_{U}$ **as an entropy related function of the independent variables in parenthesis**. Subscript phen indicates evaluation along the phenomenological path, the observable path where the independent states and dissipative process variables are available and measured at each instant. Since the product of temperature and entropy change (TdS) in Equation (5) subsumes the heat and flow transfer terms in Equations (2) and (3), Equation (5) is valid for all systems, open and closed [7,8,9,10,12]. Equation (5), which governs the system along an irreversible path, has a pair of unknowns $\delta S^{\prime }>0$ and dU; all other terms are observable and can be measured, as shown later.

To solve, a second independent equation will arise via $\delta S^{\prime }=0$ [7,8,9,10] applied to Equation (5). Recalling the thermodynamic state principle, all process paths between the same initial and end states must have the same difference between states: $dU={dU}_{irr}\;$=${\;dU}_{rev}$, $dS={dS}_{irr}={dS}_{rev}$, etc., whether the path be reversible or irreversible. Substituting difference values into Equation (5) with $\delta S^{\prime }=0$, and solving yields

(6) $$dU={dU}_{rev}=TdS_{rev}-\sum Y_{l,rev}dX_{l}\;+\sum \mu_{k,rev}dN_{k}=T{dS}_{U,rev}(S,X_{l},N_{k}),$$

where subscript *rev* indicates a quantity evaluated under reversible conditions. (Classical thermodynamics calculates entropy change along a reversible path, Equations (4) and (6), with reversible energy changes conveniently calculated via ideal response to loads, e.g., elastic deformation or non-saturating magnetics). Substituting dU =${\;dU}_{rev}$ from Equation (6) into Equation (5) and rearranging yields entropy generation

(7) $$\delta S^{\prime }=dS-\frac{\sum Y_{l}dX_{l}\;}{T}+\frac{\sum \mu_{k}dN_{k}}{T}-\frac{dU_{rev}}{T}={\delta S}_{U,phen}(S,X_{l},N_{k})-{dS}_{U,rev}(S,X_{l},N_{k})$$

for all systems. The middle equalities of Equations (5) and (6) serve to evaluate the right-side terms of Equation (7).

In Equations (3) and (4) and forthcoming entropy formulations, summation signs *∑* indicating multiple heat and mass flow terms are omitted for convenience.

### 2.2. Entropy Generation of Various System Classes via Thermodynamic Potentials

To evaluate entropy generation, Equation (7) requires the system’s changes of entropy dS and internal energy $dU_{rev}$. These are difficult to determine, especially for non-thermal and multi-component systems [16], necessitating the widely used “steady state” assumption ($dS=dU_{rev}=0$) that led to Prigogine’s stationary nonequilibrium transformation or minimum entropy generation $\delta {S^{\prime }}_{min}=\frac{PdV}{T}+\frac{\sum \mu_{k}dN_{k}}{T}$ for a reacting compressible system [5,6,12]. Via Legendre transforms, equivalent forms of Equations (5) and (7) are derived in terms of observable, measurable and more easily controllable system properties such as temperature and pressure [16] for **all systems, open or closed, steady or unsteady.** The thermodynamic potentials (with *PV* work replaced by generalized *YX* work)––enthalpy H=U+YX, Helmholtz free energy A=U–TS and Gibbs free energy G=U+YX–TS––are differentiated and solved for dU, the result of which is then inserted into Equation (5) to get:

(8a) $$T\delta S^{\prime }+dH=TdS+\sum^{l}X_{l}dY_{l}+\sum^{k}\mu_{k}dN_{k}=T{\delta S}_{H,phen}\left(S,Y_{l},N_{k}\right)$$

(8b) $$T\delta S^{\prime }+dA=-S_{A}dT-\sum^{l}Y_{l}dX_{l}+\sum^{k}\mu_{k}dN_{k}=T{\delta S}_{A,phen}(T,X_{l},N_{k})$$

(8c) $$T\delta S^{\prime }+dG=-S_{G}dT+\sum^{l}X_{l}dY_{l}+\sum^{k}\mu_{k}dN_{k}=T{\delta S}_{G,phen}(T,Y_{l},N_{k})$$

Analogous to the operations performed on Equation (5) that led to Equation (7), similar operations performed on Equations (8) and solved for $\delta S^{\prime }$ render

(9a) $$\delta S^{\prime }=dS+\frac{\sum X_{l}dY_{l}\;}{T}+\frac{\sum \mu_{k}dN_{k}}{T}-\frac{dH_{rev}}{T}={\delta S}_{H,phen}(S,Y_{l},N_{k})-{dS}_{H,rev}(S,Y_{l},N_{k})$$

(9b) $$\delta S^{\prime }=\frac{-S_{A}dT}{T}-\frac{\sum Y_{l}dX_{l}\;}{T}+\frac{\sum \mu_{k}dN_{k}}{T}-\frac{{dA}_{rev}}{T}={\delta S}_{A,phen}(T,X_{l},N_{k})-{dS}_{A,rev}(T,X_{l},N_{k})$$

(9c) $$\delta S^{\prime }=\frac{-S_{G}dT}{T}+\frac{\sum X_{l}dY_{l}\;}{T}+\frac{\sum \mu_{k}dN_{k}}{T}-\frac{dG_{rev}}{T}={\delta S}_{G,phen}(T,Y_{l},N_{k})-{dS}_{G,rev}(T,Y_{l},N_{k}).$$

Equations (7) and (9), of the form of Equation (1a), require:

- ${\delta S}_{phen}$: evaluated via the expressions of the middle equalities of Equations (5) and (8) at points over the irreversible phenomenological path defined in items 2 and 5 of Section 1.2, where salient quantities can be measured.
- ${dS}_{rev}$: evaluated as an exact differential over a reversible path between the beginning and final values of the irreversible path for ${\delta S}_{phen}$, as discussed in list items 3, 4 and 5 of Section 1.2. Here, the independent states in the parentheses of Equations (5) and (8) are linear in time, as per items 3 and 4 of Section 1.2.

### 2.3. Phenomenological Entropy Generation Theorem

The results of Equations (7) and (9) can be summarized as the

**Phenomenological Entropy Generation (PEG) Theorem:**  *Spontaneous entropy (and energy) changes along the (observable) phenomenological path is the sum of the ideal (linear, reversible transformation) entropy and the internally generated entropy, respectively:*

(1b) $${\delta S}_{phen}={dS}_{rev}+\delta S^{\prime },$$

which can be solved for entropy generation, as in Equation (1a), repeated here as

$$\delta S^{\prime }={\delta S}_{phen}-{dS}_{rev}\geq 0,$$

where, for energy extraction/decomposition or system loading, $\left({dS}_{rev}\leq {\delta S}_{phen}\right)<0$, and for energy addition/formation, $0<\left({dS}_{rev}\leq {\delta S}_{phen}\right)$. Only end state measurements of system variables (before and after process interaction) are required for evaluation of ${dS}_{rev}$ (see items 3 and 4 of Section 1.2), unlike ${\delta S}_{phen}$ which requires an instantaneous account of all active processes. Note that a system’s energy change and entropy change during a process can be negative or positive, depending on the direction of energy or entropy flow across system boundaries.

### 2.4. Phenomenological Entropy Generation Functions for Various System Classes

#### 2.4.1. Thermal Systems: Internal Energy and Enthalpy

Thermal systems include heat engines (thermal energy to mechanical work), heat pumps (mechanical work to thermal energy) and hydrocarbon fuels (chemical energy to thermal energy). Convenient for fuels and open systems is enthalpy *H* [16], which replaces volume with pressure as independent variable and measures the amount of thermal energy in a system. In a chemical reaction, change in enthalpy sums the heat absorbed or released by the reaction, by non-boundary deforming interactions, and by change in internal compositional energies. A fuel source’s heating value is its enthalpy of combustion. For heat energy, the external work terms $\frac{\sum Y_{l}dX_{l}\;}{T}$ in Equations (8a) and (7) are neglected and ${dH}_{rev}$ and ${dU}_{rev}$, the maximum/minimum theoretical thermal energies available are often specified in tables, e.g., standard enthalpy of formation of pure substances [19], standard enthalpy of reaction, or standard enthalpy of combustion or heating value of fuels [20]. The heat entropy change dS in Equations (7) and (9a) is evaluated via Equation (4) using the thermal energy balance δQ=CdT—for heat flow to or from a thermal system at uniform temperature with no heat generation—rendering ${dS}_{rev}=\frac{C_{rev}dT}{T}+\frac{{\left(u+Pv\right)}_{rev}{dN}_{e}}{T}$. For a given transformation and flow, ${dH}_{rev}$ and ${dU}_{rev}$ are constants, hence the middle set of terms of Equations (7) and (9a) are extracted to obtain the phenomenological entropy generation functions:

(10a) $${\delta S}_{U,phen}=\frac{C_{rev}dT}{T}+\frac{{\left(u+Pv\right)}_{rev}{dN}_{e}}{T}-\frac{\sum Y_{l}dX_{l}\;}{T}+\frac{\sum \mu_{k}dN_{k}}{T}$$

and

(10b) $${\delta S}_{H,phen}=\frac{C_{rev}dT}{T}+\frac{{\left(u+Pv\right)}_{rev}{dN}_{e}}{T}+\frac{\sum X_{l}dY_{l}\;}{T}+\frac{\sum \mu_{k}dN_{k}}{T}.$$

$C_{rev}$ is the heat capacity (which can be obtained at standard room temperature and pressure) and ${\left(u+Pv\right)}_{rev}$ is the open-system flow enthalpy which, in the case of evaporation, is the latent heat of vaporization. Equation (10a) applies to a thermal system doing external work or receiving non-thermal energy across its boundary; Equation (10b) to a thermal system undergoing non-boundary-deforming internal transformation. Here, $\sum \mu_{k}dN_{k}=\sum \mu_{k}\left({{dN}^{r}}_{k}+{{dN}^{d}}_{k}\right)$, the sum of the energy change due to combustion, nuclear or other exothermic/endothermic chemical reaction *r*, and diffusion *d* (as in a flame).

#### 2.4.2. Boundary-Loaded (Work-Capable) Systems: Helmholtz Potential

Most electrical, structural and mechanical systems do not use or produce *useful* thermal energy but output work and/or use an external power supply. With Equations (10a) and (10b) inadequate for these systems, the Helmholtz free energy *A* [16] replaces entropy *S* with temperature *T* as independent variable, offers an adequate, consistent and convenient characterization, and measures maximum/minimum boundary work from/to a thermodynamic system. Referring to Equation (9b), *dA_rev_* is the maximum/minimum (theoretical) work possible. During work output, energy extraction or system loading, $dT\geq 0,dX_{l}\geq 0,dN_{k}\leq 0$ and ${dA}_{rev}\leq 0$, rendering $\delta S^{\prime }\geq 0$. For work input, energy addition or product formation, $dT\leq 0,dX_{l}\leq 0,dN_{k}\geq 0$ and ${dA}_{rev}\geq 0$, reversing the respective signs of terms in Equation (9b) to accord with the second law $\delta S^{\prime }\geq 0$ [5,8,9,19]. A comparison of Equations (6) and (9b) shows that the Helmholtz relation conveniently absorbs $dU_{rev}$ and dS into $dA_{rev}$ and $-S_{A}dT$, removing the need to measure heat or mass transfer across the system boundary and the need to determine $dU_{rev}$, which is ambiguous for non-thermal systems. Most work-capable systems have a standardized maximum work $dA_{rev}$ obtainable, e.g., the elastic energy function for deformable solids. With a specified $dA_{rev}$, $\delta S^{\prime }$ in Equation (9b) measures the irreversible entropy generation pertaining to dissipation of useful energy via work across the thermodynamic boundary, which requires the instantaneous evaluation of the Helmholtz phenomenological entropy generation [8,9] terms in Equation (8b),

(10c) $${\delta S}_{A,phen}\left(T,X_{l},N_{k}\right)=\frac{\delta A_{phen}}{T}=\frac{-S_{A}dT}{T}-\frac{\sum Y_{l}dX_{l}\;}{T}+\frac{\sum \mu_{k}dN_{k}}{T},$$

where $\delta A_{phen}=-S_{A}dT-\sum Y_{l}dX_{l}\;+\sum \mu_{k}dN_{k}$. Both ${\delta S}_{H,phen}$ and ${\delta A}_{phen}$ have consistent interpretations in all boundary-loaded systems, with terms composed of conjugate pairs involving physically observable and readily measurable changes in intensive and extensive system variables *dT*, *dX_l_* and *dN_k_*. For a non-reactive non-diffusive (${{dN}^{r}}_{k}={{dN}^{d}}_{k}=0$) system, such as a lubricated mechanical interface, a fatigue-loaded component, and others, the last term of Equation (10c) can be neglected. Note that the very slow and/or (typically laboratory-controlled) isothermal case gives minimum entropy generation ${\delta S}_{min}=\frac{-\sum Y_{l}dX_{l}\;}{T}$. Various forms of work $\sum Y_{l}dX_{l}\;$ include frictional $F_{f}dx$, electrical vdq, shear Vτdγ, compression PdV, and magnetic *BdM*, among others.

#### 2.4.3. Internally Reactive Systems and Energy Storage Systems: Gibbs Potential

Reactive systems (chemical, nuclear, among others) undergo energy transformations via changes in composition. Energy storage and power sources such as batteries, nuclear power plants, and super capacitors involve changes in active species. The Gibbs free energy *G* in Equation (8c) replaces entropy *S* with temperature *T* and generalized position X with generalized force *Y* as independent variables [16], and measures maximum internal work or compositional (reactive) energy obtainable from a thermodynamic system. Equations (8c) and (9c) apply to all reactions, such as chemical formation/decomposition of substances, phase transitions, radioactive decay, etc. During active species consumption or system decomposition, $dT\geq 0,dY_{l}\leq 0,dN_{k}\leq 0$ and $dG_{rev}\leq 0$, rendering $\delta S^{\prime }\geq 0$. For active species production or system formation, $dT\leq 0,dY_{l}\geq 0,dN_{k}\geq 0$ and $dG_{rev}\geq 0$, reversing the signs of the respective terms in Equation (9c) to preserve $\delta S^{\prime }\geq 0$. With a specified $dG_{rev}$ (most energy systems have rated capacities and specific energies), Equation (9c) measures the actual irreversible entropy generation pertaining to the dissipation of useful energy via compositional changes. From Equation (8c), the Gibbs phenomenological entropy generation [7,10]

(10d) $${\delta S}_{G,phen}\left(T,Y_{l},N_{k}\right)=\frac{\delta G_{phen}}{T}=\frac{-S_{G}dT}{T}+\frac{\sum X_{l}dY_{l}\;}{T}+\frac{\sum \mu_{k}dN_{k}}{T},$$

where $\delta G_{phen}=-S_{G}dT+\sum X_{l}dY_{l}\;+\sum \mu_{k}dN_{k}$. Both ${\delta S}_{G,phen}$ and ${\delta G}_{phen}$ have consistent interpretations in all systems undergoing active compositional reactions (charge, discharge or combinations) and are composed of conjugate pairs involving physically observable and readily measurable system variables *dT*, *dY* and *dN_k_*. For constant-pressure reactive system-process interactions such as cycling of electrochemical energy systems [7,10], the term *XdY*/*T* can be neglected. For non-reactive (${dN}_{k}=0$) energy systems such as hydraulic/pressure accumulators, the last term in Equation (10d) can be dropped.

### 2.5. Generalized Material Properties, Entropy Content S, Internal Free Energy Dissipation “–SdT”

The irreversible Helmholtz and Gibbs fundamental relations, Equation (8b,c), introduced “*–SdT*”, the portion of the free energy dissipated and accumulated internally by a loaded system, typically observed as the rise in temperature of the system under non-thermal loading. This can include effects of plastic work, friction, resistive Ohmic or Joule heat, chemical reaction heat generation, and sometimes heat from an external source. Temperature change *dT* is driven by the system’s entropy content *S.* Without an entropy measurement device, *S* is often neglected or *dT* = 0, which requires experiments to be isothermal and/or significant temperature corrections for real-world applications. With δW=YdX, the energy-based Helmholtz and Gibbs equations suggest $A=A\left(T,X,N\right)$ and $G=G\left(T,Y,N\right)$. The entropy-based Massieu functions suggest $S_{A}=S_{A}\left(T,X,N\right)$ and $S_{G}=S_{G}\left(T,Y,N\right)$ wherein the entropy of a system depends on temperature *T*, generalized position X (for Helmholtz potential), generalized force *Y* (for Gibbs) and number of moles N, all of which are experimentally and instantaneously measurable. Via partial derivatives, total Helmholtz and Gibbs **entropy** changes

(11a) $${dS}_{A}={\left(\frac{\partial S_{A}}{\partial T}\right)}_{X,N}dT+{\left(\frac{\partial S_{A}}{\partial X}\right)}_{T,N}dX+{\left(\frac{\partial S_{A}}{\partial N}\right)}_{T,X}dN,$$

(11b) $${dS}_{G}={\left(\frac{\partial S_{G}}{\partial T}\right)}_{Y,N}dT+{\left(\frac{\partial S_{G}}{\partial Y}\right)}_{T,N}dY+{\left(\frac{\partial S_{G}}{\partial N}\right)}_{T,Y}dN.$$

Here, $dN={dN}_{e}+{dN}_{k}$ includes effects of mass flow and internal compositional changes/chemical reactions, respectively. From Maxwell relations and Callen’s derivatives reduction [16], Equations (11) can be re-stated using derived measurable system parameters [16,21,22], in terms of generalized work variables *X*, *Y*, as

(12) $${\left(\frac{\partial S_{A}}{\partial T}\right)}_{X,N}=\frac{C_{X}}{T};\;{\left(\frac{\partial S_{G}}{\partial T}\right)}_{Y,N}=\frac{C_{Y}}{T};\;{\left(\frac{\partial S_{A}}{\partial X}\right)}_{T,N}={\left(\frac{\partial Y}{\partial T}\right)}_{X,N}=\frac{\alpha }{\kappa_{T}}=\alpha E^{\prime }=\beta ;\;{\left(\frac{\partial S_{G}}{\partial Y}\right)}_{T,N}=-{\left(\frac{\partial X}{\partial T}\right)}_{Y,N}=-X\alpha ;\;{\left(\frac{\partial S_{A}}{\partial N}\right)}_{T,X}=-{\left(\frac{\partial \mu }{\partial T}\right)}_{X,N}=-\lambda_{X};\;{\left(\frac{\partial S_{G}}{\partial N}\right)}_{T,Y}=-{\left(\frac{\partial \mu }{\partial T}\right)}_{Y,N}=-\lambda_{Y},$$

where $C_{X}>0$ and $C_{Y}>0$ are heat capacities (for solids, $C_{X}\approx C_{Y}=C$), $\alpha =\frac{1}{X}{\left(\frac{\partial X}{\partial T}\right)}_{Y,N}>0$ is the thermal coefficient of *generalized displacement*, $\kappa_{T}=\frac{1}{E^{\prime }}-\frac{1}{X}{\left(\frac{\partial X}{\partial Y}\right)}_{T,N}>0$ is generalized “isothermal loadability” [9], obtained via a reduction of the isothermal Gibbs derivative [16] ${\left(\frac{\partial G}{\partial Y}\right)}_{T,N}=X$ to give ${\left(\frac{\partial^{2}G}{\partial Y^{2}}\right)}_{T,N}=-X\kappa_{T}$, a system/material property whose inverse defines the **load modulus** *E’* also derived from the second partial isothermal Helmholtz derivative (Appendix A). The equation $\frac{\alpha }{\kappa_{T}}=\alpha E^{\prime }=\beta >0$ is the thermal coefficient of *generalized force* (pressure, stress, voltage, etc.); $\lambda_{X}>0$ and $\lambda_{Y}>0$ are the coefficients of thermal chemico-transport decay (for solids $\lambda_{X}\approx \lambda_{Y}=\lambda$) including the combined effects of internal reaction and mass flow on entropy content.

Heat capacity *C* measures the system’s thermal response to heat transfer, retaining consistent meanings in all systems, and α measures non-thermal and non-chemical response (e.g., mechanical, electrical, etc.) to heat and temperature changes, obtained by defining *YX* for the specific system-process interaction. Generalized $\kappa_{T}$—the inverse of the load-specific modulus *E*’—represents isothermal ***loadability***, a measure of a material/system’s “cold” response to boundary loading: ***loadability*** is ***compressibility*** (inverse of bulk modulus) for a compressible system [16], ***bendability*** for a beam under bending [9], ***shearability*** (inverse of shear modulus) for shearing or torsional loading [8], and ***conductance*** (inverse of resistance) for electrical work. Derivations and detailed discussions of the properties in Equations (12) are presented in Appendix A and in reference [22] specifically for a shear-loaded system. **These formulations can be used to define new system- and process-specific material properties for assessing system/material performance and behavior**.

By substituting Equations (12) into Equations (11), we have

(13) $$dS_{A}=\frac{C_{X}}{T}dT+\beta dX-\lambda_{X}dN,\;dS_{G}=\frac{C_{Y}}{T}dT-X\alpha dY-\lambda_{Y}dN.$$

Integrating with an initial condition $S_{0}=0$ on entropy (valid in degradation analysis) gives Helmholtz and Gibbs entropy contents

(14) $$S_{A}=C_{X}lnT+\beta X-\lambda_{X}N,\;S_{G}=C_{Y}lnT-X\alpha Y-\lambda_{Y}N$$

as functions of observable and measurable system-process phenomenological variables *T*, *X*, *Y*, *N* and material properties C, α, $\kappa_{T}$, μ. Internal free energy dissipation via Helmholtz and Gibbs potentials are then

(15) $$-S_{A}dT=-\left(C_{X}lnT+\beta X-\lambda_{X}N\right)dT,-S_{G}dT=-\left(C_{Y}lnT-X\alpha Y-\lambda_{Y}N\right)dT.$$

While the system’s state response and process variables (*T*,*X*,*Y*,*N*) are directly dependent on prevalent interaction rates (e.g., strain rates, electric currents, loads, etc.) and conditions (e.g., external source heating or cooling), the material properties (*C*, *α*, $\kappa_{T}$, *λ*) can be assumed steady over a wide range of values of the state variables. Although the material properties may vary with changing state variables, the effects of such changes are minimal in a stable system with no discontinuities such as phase changes, severe chemical reactions, etc.

For compositionally changing systems via chemical, nuclear or other reactions (conveniently characterized by the Gibbs potential), an alternate formulation is the Gibbs–Duhem equation [7,10,12,16]:

(16) $$-SdT+VdP=\sum N_{k}d\mu_{k}.$$

Similar expressions can be established for other compositionally changing systems. For energy systems, the choice of Equation (16) or the second of Equations (15) depends on convenience and desired analysis output. *“-SdT”* is termed **MicroStructuroThermal** (**MST)** energy dissipation [8,9] to suggest the dissipated energies as the source of heat. For electrochemical energy systems such as batteries and capacitors—where the last terms in Equation (16) and the second of Equations (15) are expressed via Faraday’s electrolysis law in terms of cell charge capacity q and potential *v*—these equations are more specifically named **ElectroChemicoThermal** (**ECT)** energy dissipation [7,10]. An application is presented in Section 4.3.

For reactive boundary-loaded (*YdX*) systems, substitute the first of Equations (15) into Equation (10c) to give

(17a) $${\delta S}_{A,phen}\left(T,X_{l},N_{k}\right)=\frac{\delta A_{phen}}{T}=-\left(C_{X}lnT+\beta X-\lambda_{X}N\right)\frac{dT}{T}-\frac{YdX}{T}+\frac{\sum \mu_{k}{dN}_{k}}{T},$$

and for internally reactive systems under displacement-controlled or non-boundary-deforming loading *XdY*, substitute the second of Equations (15) into Equation (10d) to give

(17b) $${\delta S}_{G,phen}(T,Y_{l},N_{k})=\frac{\delta G_{phen}}{T}=-\left(C_{Y}lnT-X\alpha Y-\lambda_{Y}N\right)\frac{dT}{T}+\frac{XdY}{T}+\frac{\sum \mu_{k}{dN}_{k}}{T}.$$

Equations (17) are posed in terms of the system’s phenomenological variables, which are instantaneously measurable intensive and extensive system properties and process parameters that characterize the active phenomena along irreversible and reversible paths.

### 2.6. Stress vs. Strength Sign Conventions

For material property definitions, generalized force is interpreted as generalized **useful force, strength or potential** in line with the definitions of the free energies as maximum **useful work** obtainable from a system (Gibbs: internal work or compositional change; Helmholtz: external or boundary work). All the energy and entropy balances here accord with the IUPAC convention of representing energy leaving the system via work (and heat) as negative. In such a loaded system, *dY* is the **decrease in strength,** and hence is negative and *dX* is the increase in displacement. This accords with an expanding gas, for which pressure drops with increasing volume. However, in mechanics and other science/engineering fields that deal with solid materials, it is common to observe and use **increase in stress** as the system is loaded. In such cases, *dY* is positive. The derivations in this article and appendix consider *dY* < 0 the **decrease in strength** in a loaded system.

### 2.7. Helmholtz-Gibbs Coupling

Energy storage systems that provide boundary (external) work via direct interaction, e.g., batteries, capacitors, and pressure tanks, undergo internal changes driven by active external interaction. As such, internal phenomenological transformations can be monitored via boundary work measurements at the work transfer interface/terminal. The pressure stored in a hydraulic accumulator reduces as the accumulator provides external work, e.g., moves a weight over a distance. The phenomenological *free* energy change of an operational hydraulic accumulator can be expressed via the Gibbs potential as $\delta G_{phen}=-S_{G}dT+VdP$ or via the Helmholtz potential as ${\delta A}_{phen}=-S_{A}dT-PdV$. For electrochemical energy systems, Osara and Bryant [7,10] presented a coupling of the internal chemical/diffusion kinetics with externally measured discharge/charge energy, μdN=−Vdq, to replace the phenomenological Gibbs relation $\delta G_{phen}=-S_{G}dT+\mu dN$ with the more convenient phenomenological Helmholtz relation $\delta A_{phen}=-S_{A}dT-Vdq$. The Helmholtz and Gibbs relations for systems with interdependent internal and external interactions provides convenient characterization methods.

### 2.8. Rates

For application to time-based measurements, the phenomenological entropy generation functions of Equations (10) will be expressed in rate forms. With transport of active species into and out of an open system, flow rate ${\dot{N}}_{e}$ replacing ${dN}_{e}$, Equations (10) in rate forms become:

(18a) $${\dot{S}}_{U,phen}=\frac{C_{X}\dot{T}}{T}+\frac{\left(u+Pv\right){\dot{N}}_{e}}{T}-\frac{Y\dot{X}}{T}+\frac{\sum \mu_{k}{\dot{N}}_{k}}{T},$$

(18b) $${\dot{S}}_{H,phen}=\frac{C_{Y}\dot{T}}{T}+\frac{\left(u+Pv\right){\dot{N}}_{e}}{T}+\frac{X\dot{Y}}{T}+\frac{\sum \mu_{k}\dot{N_{k}}}{T},$$

(18c) $${\dot{S}}_{A,phen}=\frac{-S_{A}\dot{T}}{T}-\frac{Y\dot{X}}{T}+\frac{\sum \mu_{k}{\dot{N}}_{k}}{T},$$

(18d) $${\dot{S}}_{G,phen}=\frac{-S_{G}\dot{T}}{T}+\frac{X\dot{Y}}{T}+\frac{\sum \mu_{k}{\dot{N}}_{k}}{T}.$$

The dot notation represents time rate of change *d*( )/*dt*.

### 2.9. Open Systems: Pumps, Compressors, Fuel Cells, etc.

For one-dimensional flow in open systems such as pumps, compressors, turbines and heat exchangers, among others, the second right-side term in Equation (18a,b) can be approximated as ${\left({\dot{N}}_{e}h\right)}_{exit}-{\left({\dot{N}}_{e}h\right)}_{inlet}$ (the change ($u+Pv){\dot{N}}_{e}=h{\dot{N}}_{e}$ in the control volume must equal the amounts in and out). Here, h=u+Pv is the specific standard enthalpy of the flowing fluid, and the subscripts *inlet* and *exit* denote pertinent quantities at those ports. The molar flow rate ${\dot{N}}_{e}$ can be converted to mass flow rate by multiplying by molar mass. Then, the internal energy-based phenomenological entropy rate, Equation (18a), in the absence of chemical or other reactions, becomes

(19) $${\dot{S}}_{U,phen}=\frac{C_{X}\dot{T}}{T}+\frac{\sum \left[{\left({\dot{N}}_{e}h\right)}_{exit}-{\left({\dot{N}}_{e}h\right)}_{inlet}\right]}{T}-\frac{Y\dot{X}}{T},$$

where the last term can be output turbine power or input compressor/pump power.

Equation (19) can be used to monitor all boundary-loaded open systems in operation, including chemically reactive systems such as fuel cells—which have coupled boundary work, as discussed previously—simply by measuring inlet and exit flow rates, temperatures, generalized forces and velocities. Similar formulations for enthalpy can be derived, based on Equation (18b).

## 3. Evaluating Total Entropy Generation and Path (Line) Integrals

To estimate total entropy generation *S’*, $\delta S^{\prime }$ in Equation (1a) must be integrated from the initial state *o* to the final state *f* in thermodynamic state space:

(20) $$S^{\prime }=\int_{o}^{f}\left[{\delta S}_{phen}(Z,\zeta_{k})-{dS}_{rev}(Z_{rev})\right]=\int_{t_{o}}^{t_{f}}\left[{\dot{S}}_{phen}(Z,\zeta_{k})-{\dot{S}}_{rev}(Z_{rev})\right]dt,$$

where $t_{o}$ and $t_{f}$ are the times of the initial and final states, and the entropy generation functions ${\delta S}_{phen}$ and ${dS}_{rev}$ and their time rates ${\dot{S}}_{phen}$ and ${\dot{S}}_{rev}$ are defined by the middle equality functions in Equations (5) and (8). The phenomenological entropy generation function ${\delta S}_{phen}$ (or ${\dot{S}}_{phen}$) is evaluated along the irreversible *phenomenological* path where states {$Z,\zeta_{k}\}$ are measured. The reversible entropy change function ${dS}_{rev}$ (or ${\dot{S}}_{rev}$) is evaluated along the reversible path with states {$Z_{rev}\}$. Proper selection of ${\delta S}_{phen}$—the focus of Section 2—depends on system internal conditions and boundary loads.

To estimate $S^{\prime }=S^{\prime }(t)$ at time *t*, $t_{o}<$ t < $t_{f},$ replace $t_{f}$ in Equation (20) with *t* and let {$Z_{rev}(t)\}$ be the projection of {$Z(t),\zeta_{k}(t)\}$, i.e., the thermodynamic states {$Z_{rev}(t)\}$ of the reversible path are related to their counterparts {$Z(t),\zeta_{k}(t)\}$ from the phenomenological path. With this, the integrals can be estimated via the methods of item 2 of Section 1.2.

## 4. Sample (Phenomenological) Entropy Generation Calculations

Sliding of copper against steel, shearing of grease, discharge and recharge of a lithium-ion battery, fatigue of a steel rod, and flow through a pump will illustrate application of the entropy generation theory.

### 4.1. Friction Sliding of Copper against Steel at Steady Speed—(Steady State)

**Figure 1:**
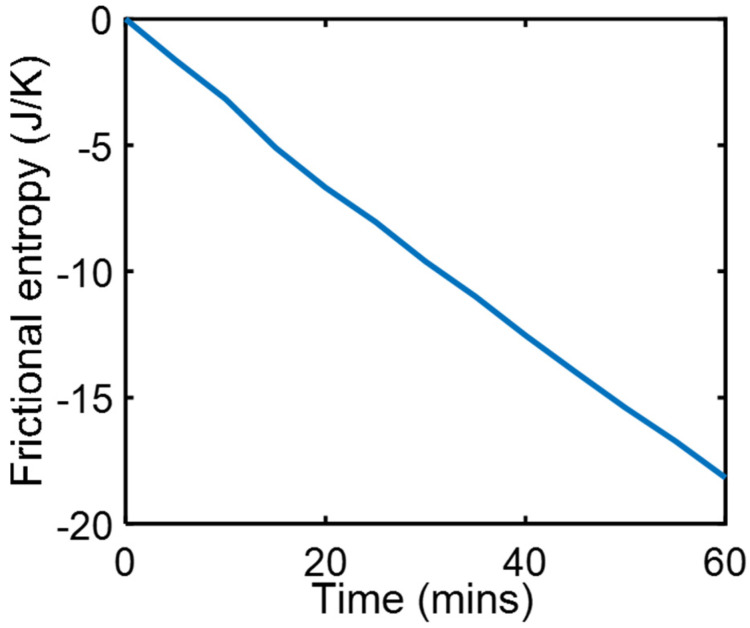
Phenomenological frictional entropy over time during boundary-lubricated frictional wear.

During a series of friction and wear tests, a copper rider pressed by 9.7 kg dead weight against a steel countersurface slid at steady speed $\dot{x}$ = 3.3 ms^−1^ under carefully maintained thermal and lubricated boundary conditions [23]. Measured were friction force F and temperatures at three locations in the copper, to estimate friction heat generation $F\dot{x}$, heat flow and surface temperature T, all of which were steady during sliding to render $\dot{T}=0$, which when substituted into Equation (18c) yields $S_{phen}^{\prime }=-\int_{t_{o}}^{t_{f}}\frac{F\dot{x}}{T}dt$. Using measured values, the integral was evaluated over the test time interval to obtain the entropy generation plot in Figure 1.

### 4.2. Mechanical Shearing of Grease—Shear Stress and Shear Strain (Helmholtz Potential)

**Figure 2:**
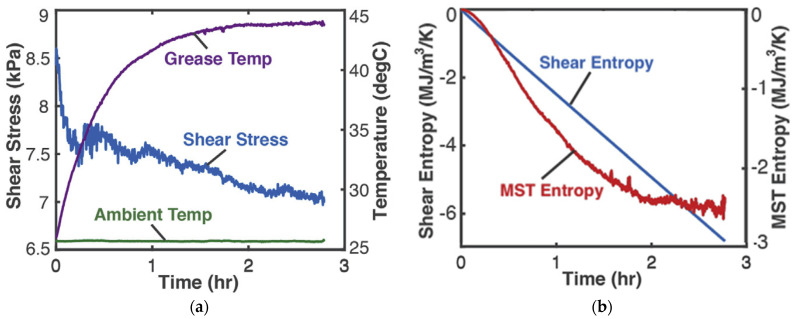
(**a**) Monitored parameters during grease shearing and (**b**) phenomenological entropy density terms: shear entropy density and MST entropy density over time [8].

0.25 kg of Aeroshell 14 aircraft NLGI 4 lithium grease in a cup was sheared by a rotating impeller. The grease-in-cup system was treated as closed and non-reacting. Tests and procedures [8] at impeller speed 3 Hz measured impeller power *M_T_ω* (the product of torque *M_T_* and rotational speed *ω*) and temperatures (via thermocouples) of grease *T* and ambient, which are plotted versus time in Figure 2a. In terms of native grease internal variables, $M_{T}\omega =V\tau \dot{\gamma }$ gives the shear power as the product of volume V, shear stress τ and shear strain rate $\dot{\gamma }$. Helmholtz entropy content *density S_A_*—the first of Equations (14) divided by *V*—was substituted into Equation (18c) with ${\dot{N}}_{k}=0,\;Y=\tau$, $\dot{X}=V\dot{\gamma }$, to yield phenomenological entropy density

(21) $$S_{phen}^{\prime }=-\int_{t_{o}}^{t_{f}}\frac{S_{A}\dot{T}}{T}dt-\int_{t_{o}}^{t_{f}}\frac{\tau \dot{\gamma }}{T}dt.$$

With the data of Figure 2a, the integrals in Equation (21) were estimated numerically via the methods of Section 1.2 to yield the entropy generation versus time plots in Figure 2b, where the MST entropy density and shear entropy density are the first and second integrals of Equation (21), respectively.

### 4.3. Discharge of Lithium-Ion Battery—Voltage and Charge (Helmholtz-Gibbs Coupling)

**Figure 3:**
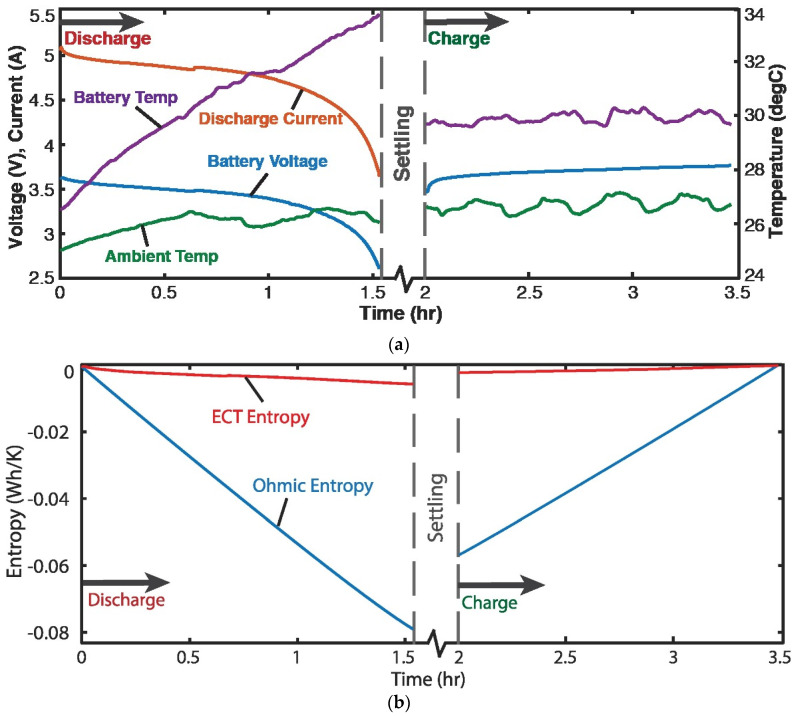
(**a**) Monitored parameters during Li-ion battery cycling showing 1.5 h discharge starting at ~5 A, followed by 1.5 h charge at 3 A. (**b**) phenomenological entropy terms: Ohmic entropy and ECT entropy over time [7].

Four 3.7 V, 11.5 Ah single-cell lithium-ion batteries were discharged at a variable discharge current of 5 A and recharged at a constant current of 3 A [7]. Figure 3a plots voltage v, current *I*, temperatures of battery *T* and ambient, measured during the battery cycle. For a battery, the Helmholtz-Gibbs coupling, Section 2.7, yields $\sum \mu_{k}{\dot{N}}_{k}=vI$, the Ohmic power. Substituting into Equation (19c) yielded

(22) $${{\dot{S}}^{\prime }}_{phen}=\frac{-S\dot{T}}{T}+\frac{vI}{T}.$$

Via the Gibbs-Duhem formulation, Equation (16), at constant pressure, $-S\dot{T}=q\dot{v}$ (applying Faraday’s electrolysis law, see paragraph after Equation (16)), substituted into Equation (22) and integrated over time rendered, for discharge and charge,

(23) $$S_{phen}^{\prime }=\int_{t_{o}}^{t_{f}}\frac{q\dot{v}}{T}dt+\int_{t_{o}}^{t_{f}}\frac{vI}{T}dt.$$

Here, $q=\int_{t_{0}}^{t_{f}}Idt$ is the charge content. With the data of Figure 3a, the integrals in Equation (23) were estimated via the methods of Section 1.2 to yield the entropy generation versus time plots in Figure 3b, where the *ElectroChemicoThermal* ECT entropy and Ohmic entropy are the first and second integrals of Equation (23), respectively.

### 4.4. Fatigue of Metals—Stress and Strain (Helmholtz Potential)

**Figure 4:**
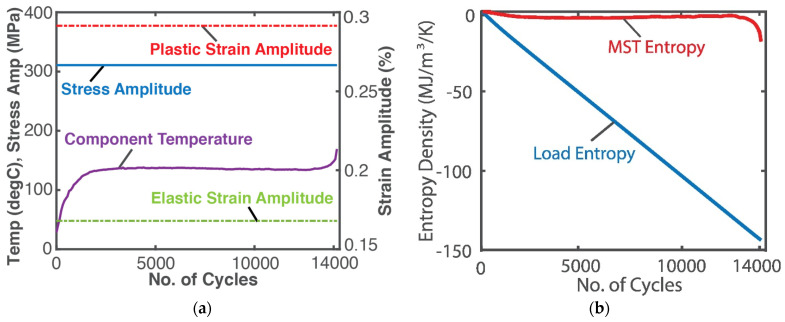
(**a**) Monitored parameters during steel rod fatigue loading and (**b**) phenomenological entropy terms: load entropy density and MST entropy density over time [9].

A high-resolution infra-red camera monitored the temperature profile of an SS 304 stainless steel rod subjected to a 10 Hz displacement-controlled cyclic bending load until fatigue failure [24]. See Figure 4a. Here, boundary work $Y\dot{X}=V\sigma :\dot{\varepsilon }$, where σ is the stress tensor and ε is the elemental strain rate tensor, both having elastic and plastic components, viz $\sigma =\sigma_{e}+\sigma_{p}$, $\varepsilon =\varepsilon_{e}+\varepsilon_{p}$. Existing models [25] estimated the stress and strain. As in the case of grease shearing, Helmholtz entropy content *density S_A_*—the first of Equations (14) divided by volume *V*—was substituted into (18c) with ${\dot{N}}_{k}=0$ to obtain

(24) $$S_{phen}^{\prime }=-\int_{t_{o}}^{t_{f}}\frac{S_{A}\dot{T}}{T}dt-\int_{t_{o}}^{t_{f}}\frac{\sigma :\dot{\varepsilon }}{T}dt.$$

Substituting stress, strain, temperature and material property values, the integrals in Equation (24) were estimated via the methods of Section 1.2 to render the entropy generation plots in Figure 4b, where the MST entropy density ${S^{\prime }}_{\mu T}$ (red plot) and load entropy density *S’_W_* (blue plot) are the first and second integrals of Equation (24), respectively. For low-cycle fatigue, with significant plastic deformation, the modulus defined in Equations (12) and (13), substituted into entropy content density *S_A_* in Equation (24), is the hardness modulus.

### 4.5. Pump Flow—Pressure and Flow Rate (Internal Energy)

**Figure 5:**
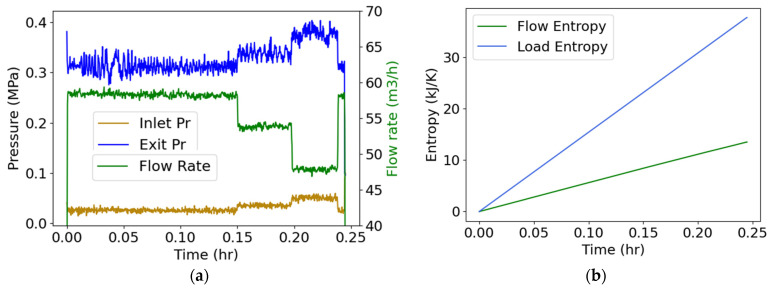
(**a**) Monitored inlet/exit pressures and flow rate during a centrifugal motor pump operation, and (**b**) phenomenological entropy terms: flow entropy and load entropy over time.

Water flowed through a three-phase 15-hp 260-gpm centrifugal motor-pump, instrumented to measure inlet and exit pump pressures and flow rate [26], which are plotted versus time in Figure 5a. A valve adjusted the flow rate. Measured volumetric flow rate was converted to mass flow rate using the density of water ($\dot{m}=\rho \dot{V}$), and pump power $Y\dot{X}=M_{T}\omega$, the product of torque $M_{T}$ and rotational speed ω. Substituting into Equation (19), with positive input power, yielded

(25) $${{\dot{S}}^{\prime }}_{phen}=\frac{C_{V}\dot{T}}{T}+\frac{\sum \left[{\left(\dot{m}h\right)}_{exit}-{\left(\dot{m}h\right)}_{inlet}\right]}{T}+\frac{M_{T}\omega }{T}.$$

With no external heat source and assuming negligible rise in flow temperature which was not measured during this test—hence *T* is constant ambient temperature—the first right-side term in Equation (25) vanished. Integrating with respect to time,

(26) $$S_{phen}^{\prime }=\int_{t_{o}}^{t_{f}}\frac{\sum \left[{\left(\dot{m}h\right)}_{exit}-{\left(\dot{m}h\right)}_{inlet}\right]}{T}dt+\int_{t_{o}}^{t_{f}}\frac{M_{T}\omega }{T}dt.$$

Substituting the data of Figure 5a, the integrals in Equation (26) yielded Figure 5b plots, where the flow entropy (measuring the change in the flow between inlet and exit, green plot) and load entropy (measuring the effect of power input into the pump, blue plot) are the first and second integrals of Equation (26), respectively.

## 5. Discussion

**Here, *reversible* implies thermodynamic reversibility: any real system undergoing a spontaneous process cannot “revert” back to its original state without work from an external source, hence is thermodynamically *irreversible*.** The reversible forms of Equations (9) (wherein $\delta S^{\prime }=0$) derived directly from Legendre transforms of entropy by Francois Massieu are called the Massieu functions [16,27]. Here, by using the irreversible form of internal energy change (Equation (5)), Equations (9) are termed **the *irreversible* Massieu functions.**

### 5.1. Phenomenology

In open thermal systems, dS in the internal energy and enthalpy Equations (7) and (9a), including the heat and mass transfer entropies, is analogous to SdT/T in the Helmholtz and Gibbs energy Equation (9a,b).

Comparing the entropy Equations (3) and (4) and the corresponding energy counterparts in Equations (5), (6) (8) and (9), via the state principle, showed that changes in entropy and energy between two states are path-independent, whether the process path is reversible or irreversible, i.e., $dS={dS}_{rev}={dS}_{irr}={\delta S}_{phen}-\delta S^{\prime }$; $dE={dE}_{rev}={dE}_{irr}={\delta E}_{phen}-T\delta S^{\prime }$, where *E* is any of internal energy *U*, enthalpy *H*, Helmholtz potential *A* or Gibbs potential *G*.

**Figure 6:**
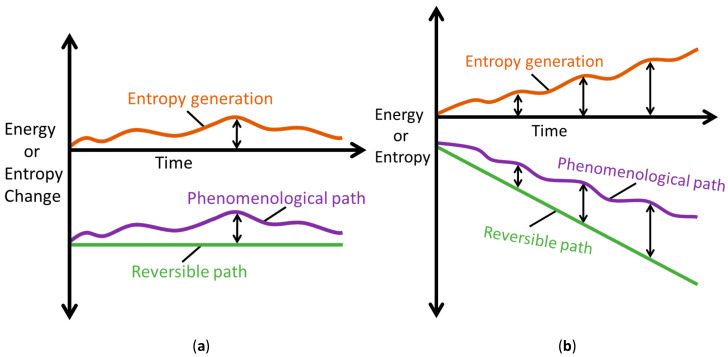
Illustrations of the *Phenomenological Entropy Generation* theorem, showing reversible (green) and phenomenological (purple) paths; the vertical difference between the paths (see black arrows) defines entropy generation or energy dissipation (orange). (**a**) Rates $dS_{rev},\;\delta S_{phen},\;\delta S^{\prime }$ and (**b**) Accumulations $S_{rev},\;S_{phen},\;S^{\prime }$.

With a transformation representable along the linear and nonlinear paths, most thermodynamic characterizations employ energy change $dE={dE}_{rev}$ and entropy change $dS={dS}_{rev}$: only end state measurements of system variables (before and after process interaction) are required for evaluation; unlike $dE={dE}_{irr}={\delta E}_{phen}-T\delta S^{\prime }$ and $dS={dS}_{irr}={\delta S}_{phen}-\delta S^{\prime }$ which require instantaneous account of all active processes. Note that a system’s energy change *dE* and entropy change *dS* during a process can be negative or positive, depending on the direction of energy or entropy flow across system boundaries, hence neither *dE* nor *dS* measures the permanent changes in the system. On the other hand, entropy generation, Equations (1a), (7) and (9), evolves monotonically as stipulated by the second law. Figure 6 depicts the *Phenomenological Entropy Generation theorem* for a loaded or spontaneously transforming system. Equation (1b), which measures the entropy generated by the system’s internal irreversibilities alone, is in accordance with experience, appearing similar to the Gouy-Stodola theorem of availability (exergy) analysis [21,28,29,30]. Rearranging Equation (1b) renders another statement of the second law or entropy balance as

(27) $$dS_{irr}={dS}_{rev}={\delta S}_{phen}-\delta S^{\prime },$$

which replaces entropy transfer in Prigogine’s entropy balance, Equation (3), with phenomenological entropy, from which entropy generation is subtracted.

As discussed previously, the *quasi*-reversible terms in the foregoing formulations proceed at constant, often standardized or predetermined rates for a given transformation, making them negligible in instantaneous energy dissipation/degradation monitoring for which the phenomenological terms are used. Table 1 summarizes phenomenological entropy generations derived in this article for various classes of open and closed systems.

### 5.2. Steady vs. Unsteady Systems: Minimum Entropy Generation (MEG) vs. MicroStructuroThermal (MST) Entropy

According to Onsager and Prigogine, the minimum entropy generation rate for a system to exist (minimally active) is the quotient of its primary work interaction (or energy transfer) and boundary temperature, i.e., ${{\dot{S}}^{\prime }}_{min}=\frac{Y\dot{X}}{T}$ [5,6,12,13]. The minimum entropy generation rates for internally reactive non-thermal open systems, including terms that characterize every significant interaction, can be derived from Equations (18a) and (19). For systems with boundary-deforming external loads, minimum entropy generation rate

(28a) $${{\dot{S}}^{\prime }}_{min}=\frac{\sum \left[{\left({\dot{N}}_{e}h\right)}_{exit}-{\left({\dot{N}}_{e}h\right)}_{inlet}\right]}{T}-\frac{Y\dot{X}}{T}+\frac{\sum \mu_{k}{\dot{N}}_{k}}{T},$$

and from (18b), for open systems without boundary-deforming work,

(28b) $${{\dot{S}}^{\prime }}_{min}=\frac{\sum \left[{\left({\dot{N}}_{e}h\right)}_{exit}-{\left({\dot{N}}_{e}h\right)}_{inlet}\right]}{T}+\frac{X\dot{Y}}{T}+\frac{\sum \mu_{k}{\dot{N}}_{k}}{T}.$$

Equations (28) only apply to steady interactions where temperature is controlled or assumed to be constant (*dT* ≈ 0). Equations (28) present the minimum conditions for simultaneous real interactions to occur (i.e., minimum perturbation from equilibrium). The steady-state frictional wear in Section 4.1 demonstrates minimum entropy generation.

For unsteady anisothermal interactions, often encountered in uncontrolled non-thermal systems (electronic, structural, mechanical, chemical, etc.), the free energies introduced the afore-named *MicroStructuroThermal* (*MST*) entropy

(29) $${{\dot{S}}^{\prime }}_{\mu T}=\frac{-S\dot{T}}{T}.$$

**Figure 7:**
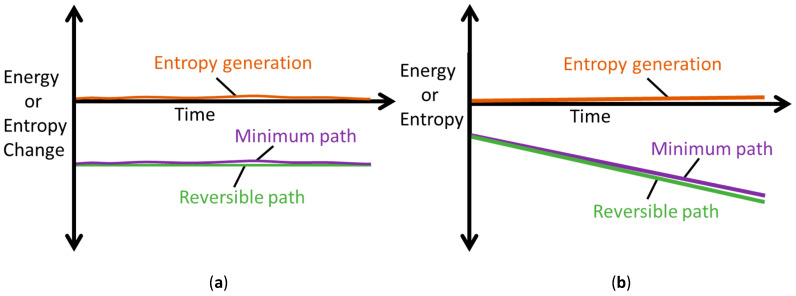
Illustrations of the Minimum Entropy Generation theorem, showing reversible (green) and minimum (purple) paths; the difference between the paths defines minimum entropy generation (orange). (**a**) Rates $dS_{rev},\;\delta S_{min},\;\delta S^{\prime }$ and (**b**) Accumulations $S_{rev},\;S_{min},\;S^{\prime }$.

The MST entropy of Equation (29) accompanies the primary interactions defined by Minimum Entropy Generation (MEG) in Equations (28). This accords with the thermodynamic state postulate [12,16,31] which requires *r* + 1 independent, intensive properties to fully specify the state of a simple system undergoing *r* primary work interactions. In non-thermal systems, the MST entropy measures the effects of energy dissipated as heat in the system, which is therefore unavailable for work. As such, the MST entropy must be minimized to decelerate degradation of non-thermal systems, the limit of which is the MEG (where ${{\dot{S}}^{\prime }}_{\mu T}=0$). Figure 7 depicts minimum entropy generation, a slight/minimal deviation from reversibility.

In the sample demonstrations in Figure 3b and Figure 4b, the ECT/MST entropy (red plots) is significantly lower than the primary interaction entropy or MEG (blue plots), which may have justified prior approaches neglecting the former via an order of magnitude analysis. However, recent works [7,8,9,10] have shown that the ECT/MST entropy, measuring the free energy dissipation, contributes significantly to degradation.

### 5.3. Dissipation Factor and Entropic Efficiency

To measure a non-thermal system’s dissipation tendencies relative to useful work output, define the dissipation factor

(30) $$J=\frac{{S^{\prime }}_{\mu T}}{{S^{\prime }}_{W}+{S^{\prime }}_{N}},$$

the ratio of the MicroStructuroThermal MST entropy ${S^{\prime }}_{\mu T}$ to the sum of boundary work ${S^{\prime }}_{W}$ and compositional change ${S^{\prime }}_{N}$ entropies, which can consistently characterize system response to dissipative mechanisms as well as multiple systems undergoing the same output/input work. A low *J* (minimal dissipation relative to available work) is desired for optimum performance and durability.

An examination of entropy Equations (7) and (9) indicates that low entropy generation ($\delta S^{\prime }\to 0$) would make more useful system energy available, the limit of which is the reversible system ($\delta S^{\prime }=0$). Define entropic efficiency

(31) $$\eta_{S^{\prime }}=\frac{{S^{\prime }}_{W}+{S^{\prime }}_{N}}{S_{rev}},$$

the ratio of the sum of boundary work and compositional change entropies to reversible entropy $S_{rev}$. With an ideal (reversible or perfect) system—for which ${S^{\prime }}_{W}+{S^{\prime }}_{N}=S_{rev}$—establishing 100% efficiency, a high $\eta_{S^{\prime }}$ is preferred for slow degradation and optimum performance. Equation (31) appears similar to the exergy-based second-law efficiency which uses reversible work and boundary work at end states (i.e., not instantaneously determined during process interaction) [31,32].

### 5.4. Thermal vs. Non-Thermal Systems—Internal Energy and Enthalpy vs. the Free Energies

Internal energy- and enthalpy-based entropy generations, Equations (7) and (9a), have an entropy change term dS, which includes heat transfer entropy, accentuating the already known suitability and convenience of internal energy and enthalpy (heat content) in characterizing primarily heat-based (or thermal) systems. The Helmholtz- and Gibbs-based entropies of Equation (9b,c) encapsulate the effects of entropy transfers in the observable evolution of the system’s internal variables (material properties and temperature). This makes the free energies and the consequent “**free entropies**” particularly convenient for non-thermal systems and interactions where heat transfer is not readily measurable and for which thermal mechanisms primarily emanate from energy dissipation. The free entropies are more consistent for assessing system transformations from observable and measurable system variables, irrespective of surrounding conditions.

Significant increase in temperature reduces available free energy via the MicroStructuroThermal MST term, see Equation (8b,c). For non-thermal (mechanical, structural, chemical, electronic, magnetic, electrical, etc.) systems, this accords with experience, further making the free entropy formulations derived from the free energies suitable for non-thermal system characterizations. Thermal systems are utilized for heat content and typically have high temperature increase rate. This indicates that a high MST component—which accompanies high thermal energy—is favorable for thermal systems. Hence, the free energies are subject to misinterpretation and not recommended for characterizing thermal systems.

## 6. Summary and Conclusions

Building on the first-principles foundations of modern irreversible thermodynamics laid by Clausius, Rayleigh, Onsager and Prigogine, this article formulated universally consistent, instantaneous entropy generations for diverse macroscopic system categories (see summary in Table 1). Presented was thermodynamic resolution of the active processes and unsteady system responses during loading using readily evaluated entropy generation. A **Phenomenological Entropy Generation (PEG)** theorem was derived and proposed, expounding the significances of the newly introduced *MicroStructuroThermal MST* entropy (or *ElectroChemicoThermal ECT* entropy for electrochemical power systems) and previously neglected reversible entropy to characteristic entropy generation. Extending and generalizing Gibbs theory of thermodynamic stability, the Clausius inequality, Rayleigh’s energy dissipation principle, Onsager’s reciprocity, and Prigogine’s entropy balance—the hallmarks of classical and modern irreversible thermodynamics [4,5,6,12,13,33]—this article, using results from recently published experimental works [7,8,9,10], demonstrated that:

- a combination of the thermodynamic potentials and the **irreversible** form of the *TdS* equation yields the ***irreversible Massieu functions***;
- steady-state systems generate entropy at a minimum rate which, for a boundary-loaded, internally reactive open system, is the sum of *boundary work/load* entropy ${S^{\prime }}_{W}$ and *compositional change* entropy ${S^{\prime }}_{N}$ rates;
- for unsteady non-thermal systems, a *microstructurothermal MST* entropy is included to characterize the accompanying instantaneous transients during system transformation;
- *phenomenological* entropy generation ${S^{\prime }}_{phen}$ is the sum of *boundary work/load* entropy ${S^{\prime }}_{W}$, *compositional change* entropy ${S^{\prime }}_{N}$ and *microstructurothermal* MST entropy ${S^{\prime }}_{\mu T}$ *(ElectroChemicoThermal ECT* entropy ${S^{\prime }}_{VT}$ for electrochemical systems);
- entropy generation is the difference between phenomenological ${S^{\prime }}_{phen}$ and reversible $S_{rev}$ entropies at every instant, named the **Phenomenological Entropy Generation (PEG) Theorem**;
- along the phenomenological path, thermodynamic and other states germane to evaluating ${S^{\prime }}_{phen}$ and $S_{rev}$ are observable (measurable), permitting evaluation of entropy generation $\delta S^{\prime }$;
- entropy generation is always non-negative in accordance with the second law, while its constituent terms ${S^{\prime }}_{phen}$ and $S_{rev}$ are directional, negative for a loaded system and positive for an energized system. This implies $\left|{S^{\prime }}_{phen}\right|\leq \left|S_{rev}\right|$ during load application/work output or active species decomposition, and $\left|{S^{\prime }}_{phen}\right|\geq \left|S_{rev}\right|$ during energization/work input or active species formation, in accordance with experience and thermodynamic laws. In plain words, measurable energy obtained from a real system is always less than the theoretical maximum/reversible energy, and measurable energy added to a real system is always more than the minimum/reversible energy. (Modulus signs indicate magnitudes only).

## Figures and Tables

**Table 1:** Summary of entropy generation formulations for various system categories, with examples. Here, subscript *e*, and superscripts *r*, *p* and *d* represent flow, reactants, products and diffusion, respectively. For thermal systems, μ is the molar enthalpy $\frac{\partial H}{\partial N}$. For other (chemical, etc.) reactive/energy systems, μ is the molar Gibbs energy or chemical potential $\frac{\partial G}{\partial N}$. More information on c, β and λ is in Section 2.5 and Appendix A.

Category	Phenomenological Entropy Generation ${\delta S}_{phen}$	Example
**Open** ***Internal Energy***	${\delta S}_{U,phen}=$ $\frac{C_{X}dT}{T}+\frac{\left(u+Pv\right)\;{dN}_{e}}{T}-\frac{\sum Y_{l}dX_{l}\;}{T}$ Equation (10a) (for a non-reacting system)	Compressor (with temperature rise and boundary work):
		${\dot{S}}_{U,phen}=\frac{C_{V}\dot{T}}{T}+\frac{\sum \left[{\left({\dot{N}}_{e}h\right)}_{exit}-{\left({\dot{N}}_{e}h\right)}_{inlet}\right]}{T}-\frac{Y\dot{X}}{T}$ (Rate form in Equation (19))
**Thermal** ***Enthalpy***	${\delta S}_{H,phen}=$ $\frac{C_{Y}dT}{T}+\frac{\sum X_{l}dY_{l}\;}{T}+\frac{\sum \mu_{k}dN_{k}}{T}$ Equation (10b) (for a closed system)	Combustion systems without boundary work:
		${\delta S}_{H,phen}=\frac{C_{p}dT}{T}+\frac{\sum \mu_{k}{{dN}^{r}}_{k}}{T}-\frac{\sum \mu_{k}{{dN}^{p}}_{k}}{T}$
**Boundary-Loaded** ***Helmholtz*** ***potential***	${\delta S}_{A,phen}=$ $\frac{-S_{A}dT}{T}-\frac{\sum Y_{l}dX_{l}\;}{T}+\frac{\sum \mu_{k}dN_{k}}{T}$ Equation (10c)	Mechanical loading, e.g., shearing without oxidation:
		${\delta S}_{A,phen}=-\left(\rho c_{\gamma }lnT+\beta \gamma \right)\frac{dT}{T}-\frac{\tau d\gamma }{T}$
**Reactive/Energy** ***Gibbs*** ***potential***	${\delta S}_{G,phen}=$ $\frac{-S_{G}dT}{T}+\frac{\sum X_{l}dY_{l}\;}{T}+\frac{\sum \mu_{k}{\dot{N}}_{k}}{T}$ Equation (10d)	Electrochemical energy systems, e.g., batteries:
		${\delta S}_{G,phen}=-\left(ClnT-\lambda_{Y}q\right)\frac{dT}{T}+\frac{\sum \mu_{k}{{dN}^{r}}_{k}}{T}-\frac{\sum \mu_{k}{{dN}^{p}}_{k}}{T}+\frac{\sum \mu_{k}{{dN}^{d}}_{k}}{T}$
		Via Gibbs-Helmholtz coupling:
		${\delta S}_{phen}=-\left(ClnT-\lambda_{X}q\right)\frac{dT}{T}+\frac{vdq}{T}$
		Via Gibbs-Duhem formulation:
		${\delta S}_{phen}=\frac{-qdv}{T}+\frac{vdq}{T}$

## Data Availability

No new data were created or analyzed in this study. Data analyzed here are available in the referenced works from which they were reproduced. Data sharing is not applicable to this article.

## Abbreviations

**Nomenclature**	**Name**	**Unit**
*A*	Helmholtz free energy	J
*B*	magnetic field	T
*C*	Heat capacity	J/K
*F*	Faraday’s constant	C/mol
*G*	Gibbs free energy	J
*I*	discharge/charge current or rate	A
*m*	mass	kg
*n*’	number of charge species	
*M*	magnetic dipole moment	J/T
*N*, *N_k_*	number of moles of substance	mol
*P*	pressure	Pa
*q*	charge	Ah
*Q*	heat	J
*R*	gas constant	J/mol·K
*S*	entropy or entropy content	J/K (Wh/K)
S	entropy generation function	J/K (Wh/K)
*S*’	entropy generation or production	J/K (Wh/K)
*t*	time	sec
*T*	temperature	°C or K
*U*	internal energy	J
*v*	voltage	V
*V*	volume	m^3^
*W*	work	J
*Z*	thermodynamic state variable	
**Symbols**		
*μ*	chemical potential	
*ζ*	phenomenological variable	
*ρ*	density	
**Subscripts & acronyms**		
*0*	initial	
*c*	specific heat capacity	
*d*	diffusion	
*e*	flow	
*f*	final	
*ECT*, *VT*	Electro-Chemico-Thermal	
*MST*, μT	Micro-Structuro-Thermal	
*rev*	reversible	
*irr*	irreversible	
*phen*	phenomenological	
*DEG*	Degradation-Entropy Generation	
*PEG*	Phenomenological Entropy Generation	
*NLGI*	National Lubricating Grease Institute	

## Appendix A. Generalized Material Properties from First Principles

System analysis is often restricted to established material properties. This appendix derives existing and new material properties for use as performance measures. These material properties measure a system’s natural response to energy changes via heat, work and/or internal reactions. Consistent interpretations of the properties are provided, independent of system and process type. Ref. [22] details an application to lubricant grease.

Entropy content *S* is determined from a combination of a system’s state variables, material properties, and process variables, as described in Section 2.5. Maxwell relations [16]—a manipulation of the three independent mixed second partial derivatives of the Gibbs and Helmholtz free energies for a pressure-volume *PV* system (e.g., compressible systems for which δW=PdV)—yield known material properties such as heat capacity, isothermal compressibility and thermal expansion coefficient. Here, we generalize the formulations to all *YX* macro-systems (including solids, liquids and gases, for which work δW=YdX [18]), hence applicable to all forms of loading. Recall that *Y* = generalized force and *dX* = generalized displacement (or deformation), hence *X* = generalized position.

Linear and steady transformation, practically achievable via slow controlled experiments, is approximated by the quasi-static assumption, for which entropy generation is minimal. Setting *δS*^‘^ ≈ 0 in Equations (8c) and (8b) for one active/reactive species (*k* = 1) yields, respectively,

(A1) $$dG=-S_{G}dT+XdY+µdN$$

and

(A2) $$dA=-S_{A}dT-YdX+µdN.$$

Equations (A1) and (A2) are suitable for determining material properties but do not describe active nonlinear and dissipative transformations encountered in typical system operation, for which *δS*^’^ *>* 0. Equations (A1) and (A2) indicate that the Gibbs free energy *G* = *G*(*T,Y,N*) is a function of temperature *T*, generalized force *Y* and amount of active species *N*, while the Helmholtz free energy *A* = *A*(*T*,*X*,*N*) is a function of temperature *T*, generalized position *X* and amount of active species *N*. Here, active species include open systems through which active matter flows and chemically reactive systems in which quantity of active matter changes.

In terms of the partial derivatives of its independent variables, the system’s total Gibbs energy change is

(A3) $$dG=\frac{\partial G}{\partial T}dT+\frac{\partial G}{\partial Y}dY+\frac{\partial G}{\partial N}dN.$$

Direct comparison of Equations (A1) and (A3) suggests

(A4) $${\left.\frac{\partial G}{\partial T}\right|}_{Y,N}=-S_{G};\;{\left.\frac{\partial G}{\partial Y}\right|}_{T,N}=X;\;{\left.\frac{\partial G}{\partial N}\right|}_{T,Y}=µ.$$

Similarly, the total Helmholtz energy change, in partial derivative terms, is

(A5) $$dA=\frac{\partial A}{\partial T}dT+\frac{\partial A}{\partial X}dX+\frac{\partial A}{\partial N}dN.$$

Direct comparison of Equations (A2) and (A5) suggests

(A6) $${\left.\frac{\partial A}{\partial T}\right|}_{X,N}=-S_{A};\;{\left.\frac{\partial A}{\partial X}\right|}_{T,N}=-Y;\;{\left.\frac{\partial A}{\partial N}\right|}_{T,X}=µ.$$

Next, partial derivatives of Equations (A4) and (A6) establish the material properties that constitute entropy contents *S_G_* and *S_A_* for evaluating the *MicroStructuroThermal* (MST) energy change ”*–SdT*” (Section 2.5).

### Appendix A.1. Heat Capacities $C_{X}$ and $C_{Y}$

The second partial derivatives of the free energies with respect to temperature define the heat capacities of a material. Substituting the first equality of Equation (A4) into the second partial derivative of Gibbs free energy with respect to temperature, we obtain

(A7) $${\left.\frac{\partial^{2}G}{\partial T^{2}}\right|}_{Y,N}=-\frac{\partial S_{G}}{\partial T}=-\frac{C_{Y}}{T}\leq 0,$$

which is rearranged to give the system’s heat capacity at constant *generalized force/strength/potential*

(A8) $$C_{Y}={\left.T\frac{\partial S_{G}}{\partial T}\right|}_{Y,N}>0,$$

defined as the amount of heat required to change the system’s temperature by 1° at constant *force*. Substituting the first equality of equation (A6) into the second partial derivative of the Helmholtz free energy with respect to temperature yields

(A9) $${\left.\frac{\partial^{2}A}{\partial T^{2}}\right|}_{X,N}=-\frac{\partial S_{A}}{\partial T}=-\frac{C_{X}}{T}\leq 0,$$

which can be rearranged to give the heat capacity at constant *generalized position/displacement*

(A10) $$C_{X}={\left.T\frac{\partial S_{A}}{\partial T}\right|}_{X,N}>0,$$

defined as the amount of heat required to change the system’s temperature by 1° at constant *position/displacement.* As is the case for most fluids and solids, $C_{X}$ ≈ $C_{Y}$ over a wide temperature range. The heat capacities measure the system’s natural and characteristic response to heat via temperature change, and have universally consistent meaning in all materials. Equations (A8) and (A10) imply that the system entropy content *S*, *S_G_* or *S_A_* must increase in response to increase in temperature for the system to remain stable. When the entropy increase is the result of heat transfer δQ into the system only, approximated by slowly heating the stationary system, Equations (A8) and (A10) are combined and rewritten for practical purposes as

(A11) $$C_{X\;or\;Y}={\left.\frac{\delta Q}{\partial T}\right|}_{X\;or\;Y,N}>0.$$

For a pressure-volume *PV* system, $C_{X}$ = $C_{V}$ and $C_{Y}$ = $C_{P}$. The specific heat capacity *c = C/m*, where *m* is mass, is used as a material property.

### Appendix A.2. Isothermal Loadability κ_T_ and Load Modulus E’

The second partial derivative of the Gibbs free energy with respect to *generalized force Y* defines the isothermal loadability *κ_T_* of a material/system. Substituting the second equality of Equation (A4) into the second partial Gibbs derivative gives

(A12) $${\left.\frac{\partial^{2}G}{\partial Y^{2}}\right|}_{T,N}={\left.\frac{\partial X}{\partial Y}\right|}_{T,N}=-X\kappa_{T}\leq 0,$$

rearranged to obtain the material’s isothermal loadability

(A13) $$\kappa_{T}=-\frac{1}{X}{\left.\frac{\partial X}{\partial Y}\right|}_{T,N}>0,$$

hereby defined as the "cold” mechanical response to load, i.e., *generalized position/displacement* response to *generalized force* at constant temperature. Widely used in compressive systems is the isothermal compressibility which relates volume response to pressure *load*. The second partial derivative of the Helmholtz free energy with respect to *generalized position/displacement X* defines the load (or strength) modulus *E*^’^ of a material. Substituting the second equality of Equation (A6) into the second partial Helmholtz derivative yields

(A14) $${\left.\frac{\partial^{2}A}{\partial X^{2}}\right|}_{T,N}=-{\left.\frac{\partial Y}{\partial X}\right|}_{T,N}=\frac{E^{\prime }}{X}\geq 0,$$

rearranged to give the material’s isothermal load modulus

(A15) $$E^{\prime }=-X{\left.\frac{\partial Y}{\partial X}\right|}_{T,N}>0,$$

the *generalized force/strength* response to *generalized position/displacement*, commonly used in analysis of systems under external boundary load, such as stress-loaded (elastic, shear, hardness moduli) and compressible systems (bulk modulus). A comparison of Equations (A13) and (A15) shows that $E^{\prime }=\frac{1}{\kappa_{T}}$, i.e., the load modulus is the inverse of the isothermal loadability (as the bulk modulus is the inverse of the isothermal compressibility in compressible systems).

According with the sign convention for a loaded system discussed in Section 2.6, the negative signs in Equations (A13) and (A15) indicate that an increase in generalized displacement *X* corresponds to a decrease in generalized strength/force *Y*, a stability condition for all *loadable*/*work-capable* systems. For example, as a compressible system expands (energy extraction or system loading), its accumulated/internal pressure decreases, i.e., ∂V>0 ⇒∂P < 0, and vice versa for compression (energy addition), to give $\left(\frac{\partial P}{\partial V}\right)<0$. In mechanical systems, loading reduces component strength or toughness, ∂ε>0 ⇒ ∂σ < 0 to give $\left(\frac{\partial \sigma }{\partial \varepsilon }\right)<0$. In electrochemical systems such as batteries, discharge (energy extraction via charge transfer) reduces electrochemical potential or voltage, dq>0 ⇒ dV < 0 yielding $\left(\frac{\partial V}{\partial q}\right)<0$.

The loadability or modulus of most solids and liquids (the latter, to much less extent) is not significantly affected by temperature over a wide temperature range, yielding $\kappa_{T}\approx \kappa$ for most practical purposes.

### Appendix A.3. Thermal Displacement Coefficient α and Thermal Force (Strength) Coefficient β

When the heat generated in or transferred to the system raises the system’s temperature (the amount of heat required determined by the system’s heat capacity), the system’s microstructure responds to the increasing temperature (in the absence of other external/internal load). For materials that expand/contract in response to temperature change, the thermal expansion coefficient quantifies the degree of expansion/contraction. For stress-strain systems, this property is termed the *thermal strain coefficient* [8,9,22], to indicate a microstructural strain response of the system to temperature change at constant stress.

The mixed second partial derivative of the Gibbs free energy with respect to temperature *T* and *generalized force Y* defines the thermal *displacement* coefficient *α* of the system. Substituting the first and second equalities of Equation (A4) into the mixed second partial Gibbs derivative renders

(A16) $${\left.\frac{\partial^{2}G}{\partial T\partial Y}\right|}_{N}=-{\left.\frac{\partial S_{G}}{\partial Y}\right|}_{T,N}={\left.\frac{\partial X}{\partial T}\right|}_{Y,N}=X\alpha \geq 0,$$

rearranged to yield the thermal displacement coefficient

(A17) $$\alpha =\frac{1}{X}\frac{\partial X}{\partial T}>0,$$

defined for a system as the displacement induced in the system by an increase in temperature at constant *force/strength*. The thermal displacement coefficient *α* measures the system’s natural and characteristic mechanical response to temperature change via displacement change or deformation, and has universally consistent meaning in all systems. Equation (A16) implies that the system’s Gibbs entropy content *S_G_* must increase in response to decrease in generalized force *Y* for process continuity and system stability. It also establishes that the position/displacement *X* in the system spontaneously increases (e.g., expansion, strain, etc.) with increase in temperature for stability and process continuity.

The second partial derivative of the Helmholtz free energy with respect to temperature *T* and *generalized displacement X* defines the thermal *force (strength)* coefficient *β*. Substituting the first and second equalities of Equation (A6) into the mixed second partial Helmholtz derivative yields

(A18) $${\left.\frac{\partial^{2}A}{\partial T\partial X}\right|}_{N}=-{\left.\frac{\partial S_{A}}{\partial X}\right|}_{T,N}=-{\left.\frac{\partial Y}{\partial T}\right|}_{X,N}=\frac{{\left.\frac{\partial X}{\partial T}\right\rceil }_{Y}}{{\left.\frac{\partial X}{\partial Y}\right\rceil }_{T}}=-\frac{\alpha }{\kappa_{T}}=-\alpha E^{\prime }\leq 0,$$

rearranged to give the (solid) system’s thermal force (strength) coefficient

(A19) $$\beta =\frac{\alpha }{\kappa_{T}}=\alpha E^{\prime }>0,$$

the ratio of the thermal displacement coefficient to the isothermal loadability or the product of the thermal displacement coefficient and the load modulus. *β* is the *force*/*strength/stress* induced by a degree rise in temperature at constant *displacement*. For stress-strain systems, *β* is also called the thermal stress coefficient [22]. Equation (A18) implies that the system’s Helmholtz entropy content must increase in response to increase in *displacement* (or deformation, expansion, etc.) for process continuity. In general, a solid material’s strength spontaneously decreases with increase in temperature, ∂Y/∂T<0, whereas for fluids, ∂Y/∂T>0 due to the increase in the useful kinetic and thermal energy of fluids with temperature.

### Appendix A.4. Isothermal Chemical Resistances $R_{N_{Y}}$ and $R_{N_{X}}$

The second partial derivative of the Gibbs free energy with respect to number of active/reactive moles *N* defines the isothermal chemical resistance at constant *force/strength* $R_{N_{Y}}$. Substituting the third equality of Equation (A4) into the second partial Gibbs derivative yields

(A20) $${\left.\frac{\partial^{2}G}{\partial N^{2}}\right|}_{T,Y}={\left.\frac{\partial \mu }{\partial N}\right|}_{T,Y}=R_{N_{Y}}\geq 0,$$

rearranged to obtain a system’s isothermal chemical resistance at constant force

(A21) $$R_{N_{Y}}={\left.\frac{\partial \mu }{\partial N}\right|}_{T,Y}>0,$$

which measures the change in the system’s chemical (nuclear, etc.) potential with change in amount of the reactive constituent in the system. The chemical resistance is named similar to the electrical resistance due to the similarity in their fundamental formulations (in electrical/electrochemical systems, Equation (A21) is Ohm’s law *R* = *V*/*I*).

Similarly, substituting the third equality of Equation (A6) into the second partial Helmholtz derivative yields

(A22) $${\left.\frac{\partial^{2}A}{\partial N^{2}}\right|}_{T,X}={\left.\frac{\partial \mu }{\partial N}\right|}_{T,X}=R_{N_{X}}\geq 0,$$

rearranged to give the system’s isothermal chemical resistance at constant position/displacement

(A23) $$R_{N_{X}}={\left.\frac{\partial \mu }{\partial N}\right|}_{T,X}>0.$$

As with heat capacities, modulus and loadability, $R_{N_{X}}$ ≈ $R_{N_{Y}}$ for most solids and liquids at low forces and displacements.

### Appendix A.5. Thermal Chemico-Transport Decay Coefficients $\lambda_{Y}$ and $\lambda_{X}$

Substituting the first and third equalities of Equation (A4) into the mixed second partial Gibbs derivative with respect to temperature *T* and number of active/reactive moles *N* renders

(A24) $${\left.\frac{\partial^{2}G}{\partial T\partial N}\right|}_{Y}=-{\left.\frac{\partial S_{G}}{\partial N}\right|}_{T,Y}={\left.\frac{\partial \mu }{\partial T}\right|}_{Y,N}=\lambda_{Y}\geq 0,$$

rearranged to obtain the constant-force thermal (chemico-transport) decay coefficient

(A25) $${\left.\lambda_{Y}=\frac{\partial \mu }{\partial T}\right|}_{Y}>0,$$

here defined as the chemical potential response to temperature change at constant strength/force. Substituting the first and third equalities of Equation (A6) into the mixed second partial Helmholtz derivative with respect to *T* and *N* yields

(A26) $${\left.\frac{\partial^{2}A}{\partial T\partial N}\right|}_{X}=-{\left.\frac{\partial S_{A}}{\partial N}\right|}_{T,X}={\left.\frac{\partial \mu }{\partial T}\right|}_{X,N}=\lambda_{X}\geq 0,$$

rearranged to give the system’s constant-displacement thermal decay coefficient

(A27) $${\left.\lambda_{X}=\frac{\partial \mu }{\partial T}\right|}_{X}>0.$$

As observed for heat capacities and chemical resistances, at low forces and displacements and negligible variations of other properties, $\lambda_{X}$ *≈* $\lambda_{Y}$ = λ for solids and liquids over a considerable temperature range.

The conditions for system stability and/or process continuity defined by the above formulations (using the inequalities) obtain from the fundamental—or Gibbs—thermodynamic stability theory which establishes a monotonic spontaneous evolution (decline) towards stable equilibrium.

### Appendix A.6. Gibbs Properties vs. Helmholtz Properties

Table A1 lists and categorizes all the properties derived herein according to the prevalent interaction(s) they characterize.

**Table A1:** The Gibbs and Helmholtz potential-based material properties derived in Appendix A.

Category	Gibbs-Based	Helmholtz-Based
**Thermal**	Constant-Force Heat Capacity $C_{Y}={\left.\frac{\delta Q}{\partial T}\right|}_{Y,N}$	Constant-Displacement Heat Capacity $C_{X}={\left.\frac{\delta Q}{\partial T}\right|}_{X,N}$
**Thermo-mechanical**	Thermal Displacement Coefficient	Thermal Force Coefficient
	$\alpha =\frac{1}{X}\frac{\partial X}{\partial T}$	β=αE’
**Mechanical**	Isothermal Loadability	Load Modulus
	$\kappa_{T}=-\frac{1}{X}{\left.\frac{\partial X}{\partial Y}\right|}_{T,N}$	$E^{\prime }=-X{\left.\frac{\partial Y}{\partial X}\right|}_{T,N}$
**Chemical**	Isothermal Constant-Force Chemical Resistance $R_{N_{Y}}={\left.\frac{\partial \mu }{\partial N}\right|}_{T,Y}$	Isothermal Constant-Displacement Chemical Resistance $R_{N_{X}}={\left.\frac{\partial \mu }{\partial N}\right|}_{T,X}$
**Thermo-chemical**	Constant- Force Thermal Decay Coefficient	Constant-Displacement Thermal Decay Coefficient
	${\left.\lambda_{Y}=\frac{\partial \mu }{\partial T}\right|}_{Y,N}$	${\left.\lambda_{X}=\frac{\partial \mu }{\partial T}\right|}_{X,N}$

Table A2 summarizes the generic interpretations of the material properties.

**Table A2:** Material properties and their generic physical interpretations.

Property	Physical Interpretation
Heat Capacity	Material’s ability to withstand heat without an increase in temperature.
Thermal Displacement/Force Coefficient	Impact of temperature on displacement/force. Physical response to temperature change.
Load Modulus	Physical response to non-thermal load.
Chemical Resistance	Resistance to chemical conversion of reactive species.
Thermal Decay Coefficient	Chemical response to temperature change.

The foregoing formulations have been applied to grease [22], deriving and experimentally measuring new and existing grease properties for characterizing grease performance and degradation.
